# Iridium(III) Complexes Targeting Apoptotic Cell Death in Cancer Cells

**DOI:** 10.3390/molecules24152739

**Published:** 2019-07-28

**Authors:** Dik-Lung Ma, Chun Wu, Ke-Jia Wu, Chung-Hang Leung

**Affiliations:** 1 Department of Chemistry, Faculty of Science, Hong Kong Baptist University, Kowloon, Hong Kong SAR 999077, China; 2 State Key Laboratory of Quality Research in Chinese Medicine, Institute of Chinese Medical Sciences, University of Macau, Macau, Macau SAR 999078, China

**Keywords:** iridium(III) complex, apoptosis, mitochondria, cancer

## Abstract

Targeting apoptosis is a principal strategy in the design of anticancer drugs. In recent years, non-platinum-based scaffolds have been exploited as viable candidates for the exploitation of anticancer agents with potentially lower toxicity than the widely used cisplatin analogues. This review highlights the latest advances in developing iridium(III) complexes as anticancer agents that act particularly via targeting apoptotic cell death in cancer cells.

## 1. Introduction

Apoptosis, also known as programmed cell death, refers to the natural and regulated process of self-destruction by specific cells. Apoptosis is often defective in cancer cells, and this deficiency can drive the development of cancer at various phases ranging from transformation to metastasis [1]. Hence, targeting apoptosis is considered as a viable approach toward designing new anticancer therapeutics. The classic routes of apoptosis are generally categorized into mitochondria-interceded intrinsic and receptor-interceded extrinsic pathways, which are activated by a variety of mitochondrial stimuli or death-involved signal receptors, respectively. The mitochondrial pathway of apoptosis plays a multifunctional and central role in causing the death or destruction in cancer cells. Consequently, many novel therapies have been exploited to specifically engage mitochondrial apoptosis in cancer cells.

Ever since the discovery of cisplatin, inorganic medicines have remained at the forefront of chemotherapy research. However, the commonly used platinum-based therapies, including cisplatin, oxaliplatin, or carboplatin, are associated with a range of serious adverse effects. Nephrotoxicity is the most commonly specific adverse effect encountered in cisplatin patients, while myelosuppression and neurotoxicity have been linked to the use of carboplatin and oxaliplatin, respectively [2,3]. This has driven the search for other metal-based anticancer agents that retain the potency of the platinum drugs but possess fewer side effects. To date, a variety of non-platinum transition metal-based complexes, including osmium, gold, ruthenium, rhodium, and palladium complexes, have been intensively exploited for their potential use as anticancer drugs [4,5,6,7,8,9,10,11].

Recently, iridium-based compounds have drawn strong interest in view of their strong anticancer activities, versatile photophysical properties, and limited side effects. The ligand substitution kinetics of iridium compounds are several orders of magnitude larger than those of platinum scaffolds. The ease of the modification in the peripheral ligands of iridium complexes also grants them a rich diversity of molecular architectures over the typical square-planar architectures of platinum complexes, thus enabling a diverse range of bioactivities. One clear advantage for using iridium complexes as anticancer agents is their ability to be exploited for simultaneous imaging applications due to their unique spectroscopic properties. The wide and tunable emission range of iridium complexes, particularly in the important red region, enables the development of dual-functional anticancer agents and luminescent probes with a multi-color detection mode. In this review, we will discuss the latest advances in developing iridium(III) complexes as anticancer agents, with a specific focus on the capability of these compounds to induce apoptosis in cancer cells.

## 2. Mechanism of Apoptosis-Targeted Cell Death in Cancer Cells

The classic routes of apoptosis are generally categorized into mitochondria-mediated intrinsic and receptor–mediated extrinsic pathways, which are activated by a variety of mitochondrial stimuli or death-involved signal receptors, respectively [12]. The mitochondria-mediated pathway serves as a central checkpoint in the regulation of apoptosis by integrating diverse exogenous stimuli (e.g., xenobiotic or envelope polysaccharides/proteins from viruses), endogenous ions (e.g., Na^+^, K^+^, Mg^2+^, Ca^2+^, and cytoplasm/organellar-originated protons), metabolic signaling macromolecules (e.g., caspase family members, Bcl-2 family proteins, and protein kinases) as well as metabolic signaling small molecules (e.g., lipid second messengers, glutathione, nicotinamide adenine dinucleotide (NAD), nicotinamide adenine dinucleotide phosphate (NADP), adenosine diphosphate (ADP), adenosine triphosphate (ATP), and reactive oxygen species (ROS)). When death-inducing signals overwhelm life-preserving signals, mitochondrial membrane permeabilization ensues [13]. The levels of these apoptosis-inducing signals are hence critical for the regulation of this process. However, despite the different action mechanisms of mitochondria-mediated intrinsic and receptor-mediated extrinsic pathways, the cross-talk between them was extensively demonstrated in the apoptosis process [14].

### 2.1. B-Cell Lymphoma 2 (Bcl-2) Family Proteins

Bcl-2 family members are responsible for the maintenance of mitochondria integrity. The Bcl-2 family proteins cover two functionally reversed groups, including the pro-apoptotic proteins (e.g., Bim, Bad, Bak, and Bax) and the anti-apoptotic proteins (e.g., Bcl-w, Bcl-XL, and Bcl-2) that, respectively, promote and inhibit the execution of apoptosis. Bcl-2 proteins share a homologous sequence in Bcl-2 homology (BH) domains. For example, pro-apoptotic proteins can be classified into a BH3-only subfamily (e.g., Bad, Bim, and Bid) and a multidomain subfamily sharing homologous domains known as BH1–3 (e.g., Bok, Bak, and Bax). Likewise, homologous BH1–4 domains are found in anti-apoptotic proteins such as Bcl-XL and Bcl-2. The relative sensitivity of cancer and healthy cells to apoptosis-triggering media is determined by the coordination in the expression levels of the mitochondrial membrane-located anti-apoptotic proteins and transferable pro-apoptotic proteins in the mitochondria [15].

### 2.2. Caspase Family Proteins

Caspases are cysteinyl aspartic acid-specific proteases and function by cleaving through the carboxyl moiety of the aspartate residues in their target proteins. Dysfunction in the maintenance of normal caspase levels may lead to disequilibrium in physiological metabolism processes at the biochemical level, finally resulting in apoptotic cell death [16].

### 2.3. Protein Kinase Pathways

Protein kinases have also been widely studied for their role in apoptosis. Protein kinases can regulate the promotion or inhibition of apoptosis by the triggering of phosphorylation cascades of apoptosis-related proteins. Various protein kinases have been demonstrated to exert a crucial role in the apoptosis process, such as mitogen-activated protein kinases (MAPKs), kappa-B-kinase inhibitors (IKK), protein kinase-C (PKC), phosphatidylinositol 3-kinase (PI3K)/Akt, and tyrosine kinases [17].

### 2.4. Extrinsic Apoptosis Pathway

The extrinsic apoptosis pathway is initiated and further propagated upon the activation of the signal receptors located on the surface of targeted cells. Tumor necrosis factor receptor (TNFR) is a central subfamily responsible for the regulation of apoptotic processes. In the case of tumor necrosis factor alpha (TNF-α)-triggered apoptosis, a death-activation signaling complex can be generated upon the targeting binding of TNF-α to its membrane-surface-located TNFR [18].

### 2.5. Intrinsic Apoptosis Pathway

The intrinsic apoptosis pathway is mainly activated by the stimulation of chemical and/or environmental stress compounds such as H_2_O_2_, ultraviolet radiation, and ionizing radiation. The polarization status of the membrane or oxidative stress in cellular environment can be readily altered in this process. In the case of H_2_O_2_-induced apoptosis, the production of ROS is sometimes accompanied by the depolarization of the mitochondrial membrane. Afterward, further fluctuations in membrane permeability can be generated, thereby leading to the effective release of cytochrome c (Cyt-c) in mitochondria. The release of Cyt-c triggers the interference of the electron transportation status and further activates the generation of apoptosis-initiating O_2_^−^ molecules [18].

## 3. Dilemma of Conventional Apoptotic Cell-Targeted Anticancer Agents

Conventional apoptotic cell-targeted anticancer agents, such as paclitaxel, cisplatin, doxorubicin, or etoposide, activate apoptotic cell death by various pathways through interfering with the balance of energy/redox states, the expression levels of p53 proteins, the maintenance of the GD3/ceramide pathway, and the binding affinity of CD95/CD95L-specific interaction. A perturbation in the metabolism process of pro-apoptotic second messengers can be triggered, leading to the changes in permeabilization ability. However, conventional anticancer agents rely on the endogenous apoptosis-activation processes that have a high possibility of being compromised during their action. For example, apoptosis can be unexpectedly perturbed by the upregulation of Bcl-2-like signals, the block of the specific binding manner between CD95 and CD95L, the increase of antioxidant activity, and the mutation of p53 proteins. Hence, one feasible alternative strategy to execute targeted apoptosis is to activate downstream events. For example, apoptosis can be successfully achieved by the timely application of adenovirus-involved transportation of caspase family members. Alternatively, targeted apoptosis can be also achieved by bypassing the dependence of upstream signal molecules. For example, the application of anticancer agents such as CD437 and arsenite is able to trigger the apoptosis process even in the presence of caspase inhibitors in a p53-independent fashion. Likewise, a caspase-independent apoptosis process can also be induced by the application of anticancer agents (e.g., viral protein R and betulinic) by mediating the permeabilization states of mitochondrial membranes. In this case, the resistance toward conventional anticancer agents can be alleviated or even eliminated [19].

## 4. Characteristics of Transition Metal-Based Compounds

Given the variety of metal centers and ligands with different electronic and steric features available, complexes can be designed and modified to give a wide variety of specific chemical and biological properties [20,21]. Compared with organic molecules, metal complexes demonstrate a higher structural diversity due to their ability to exploit a broad variety of coordination geometries, variable bond distances, bond angles, and coordination sites. Owing to their capability to coordinate ligands in a three-dimensional configuration, metal complexes with functionalized groups can be optimized to recognize specific target biomolecules via shape interactions.

Transition metal complexes can be prepared as neutral, cationic, or anionic species depending on the nature of the conjugated ligands. This can facilitate the recognition of biomolecules via electrostatic or even coordinative interactions. For example, cationic complex ions will preferentially interact with negatively charged biomolecules and, depending on the coordination geometry available and the other ligands present in the complex, may even allow for the formation of strong coordinate bonds with negatively-charged groups in the molecule. Another distinctive property of the transition metal complex is its redox activity and the ability to cycle through multiple oxidation states. This can be exploited for developing redox-active anticancer therapeutics [22,23].

## 5. Iridium(III) Complexes with Anticancer Activity

The accidental discovery of the antiproliferative effect of cisplatin drove the development of different platinum-based drugs, including oxaliplatin and carboplatin, over the next several decades. However, despite the success of platinum-based drugs, they have been linked to serious side effects and drug resistance issues, which restricts their more widespread use. Hence, other transition metal-based compounds including ruthenium, rhodium, gold, osmium, and iridium have emerged as potential candidates for developing chemotherapeutic agents [24]. This review focuses on the recent advances of iridium(III) complexes as anticancer agents, particularly those targeting the mitochondrial apoptotic pathway.

### 5.1. Development of Organometallic Half-Sandwich Iridium(III) Compounds as Antitumor Agents

In recent years, organoiridium(III) “half-sandwich” complexes have been demonstrated to show significant anticancer activity [25,26,27,28]. The presence of cyclopentadienyl or other carbon-bound π-bond arene ligands confers an interesting hydrophilicity–hydrophobicity balance on the opposite faces of half-sandwich metal complexes, which can have strong impacts on cellular uptake and targeting [29,30,31].

**Figure 1:**
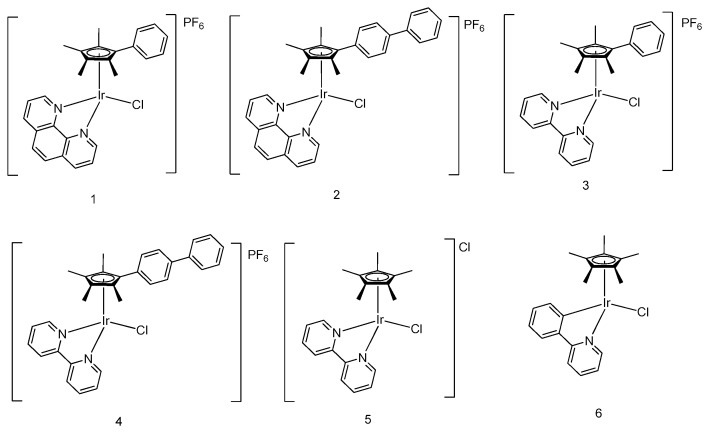
Organometallic half-sandwich iridium(III) compounds with different arene and bidentate groups.

Sadler and coworkers have reported a number of pentamethylcyclopentadienyl (Cp*)-containing iridium(III) complexes with potent antitumor effects. Subtle changes in the chemical structure of the ligands were found to produce significant changes in the anticancer activity of the scaffolds. Potency was enhanced prominently by the insertion of substituent biphenyl and phenyl moieties on the Cp* ligand of iridium(III) complexes (**1**–**4**) [32] (Figure 1). Complexes **2** and **4**, bearing the tetramethyl(biphenyl)cyclopentadienyl (Cpxbiph) ligand, showed values of IC_50_ of 0.72 μM and 0.57 μM, respectively, in human ovarian cancerous cells (A2780) in a sulforhodamine B (SRB) cell viability assay, rendering them 10 times as potent compared with their corresponding complexes **1** and **3** bearing the tetramethyl(phenyl) cyclopentadienyl (Cpxph) ligand. Moreover, they were twice as effective as cisplatin (IC_50_ = 1.2 μM). This suggested that the number of phenyl rings in the Cp ligand increase could enhance cytotoxicity activity. Mechanistically, complexes **1**–**4** were thought to induce cytotoxicity toward A2780 cells by forming Ir–DNA (where DNA refers to deoxyribonucleic acid) adducts in primer extension foot-printing assay, particularly upon the interaction with 9-ethylguanine rather than 9-ethyladenine as revealed by ^1^H NMR spectra.

Moreover, the displacement of the neutral N,N-bound-chelating ligand bipyridine (bpy) of iridium(III) complex **5** by the negatively charged C,N-bound-chelating 2-phenylpyridine (ppy) ligand in complex **6** also led to an increase in cytotoxicity activity [33]. Complex **5** displayed insignificant inhibitory effect (IC_50_ > 100 μM in SRB assay) against A2780 cells, whereas complex **6** showed an IC_50_ value of 10.8 μM, making it similar in potency to carboplatin. This suggests that the cytotoxic effect of these complexes in cancer cells also correlates with their hydrophobicity and strength of nucleobase binding. Complexes **5** and **6** both displayed a strong binding mode toward 9-ethylguanine, whereas a moderate binding mode toward 9-ethyladenine in MeOD-*d4*/D_2_O (v/v, 1:4) as revealed by ^1^H NMR spectra, rendering a more preferable interaction with 9-ethylguanine when in competition with 9-ethyladenine.

**Figure 2:**
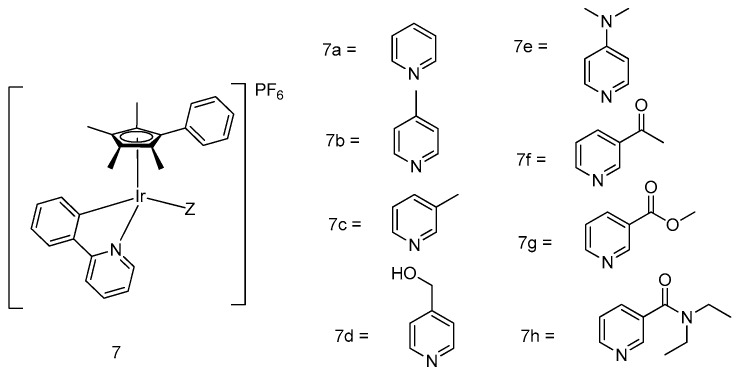
Varying the monodentate ligand in organometallic half-sandwich iridium(III) compounds.

The electronic effect on the monodentate ligand in the organometallic half-sandwich iridium(III)-centered complexes was further explored in Sadler and coworkers’ subsequent study [34]. A library of eight half-sandwich iridium(III)-conjugated complexes [(η5-Cpxph)Ir(phpy)Z]PF_6_ (**7a**–**h**) (Figure 2), where Z = pyridine or its derivatives, was successfully prepared and evaluated for cytotoxicity against various cancer cell lines. All compounds showed promising cytotoxic effect in the SRB assay against human ovarian cancer cells (A2780), human breast cancer cells (MCF-7), and human lung cancer cells (A549). The most potent candidate scaffold, complex **7e**, showed an IC_50_ value of 0.20 μM in MCF-7 cells, making it over 30 times more potent than cisplatin. From this study, it was observed that electron-withdrawing groups (acetyl, carboxylate, amido) in the pyridine ligand decreased antiproliferation activity in cancer cells. Interestingly, the active complexes also induced significant changes of the membrane potential as well as a drastic increase in ROS levels in A2780 cells, suggesting that they may be acting via mitochondrial dysfunction.

**Figure 3:**
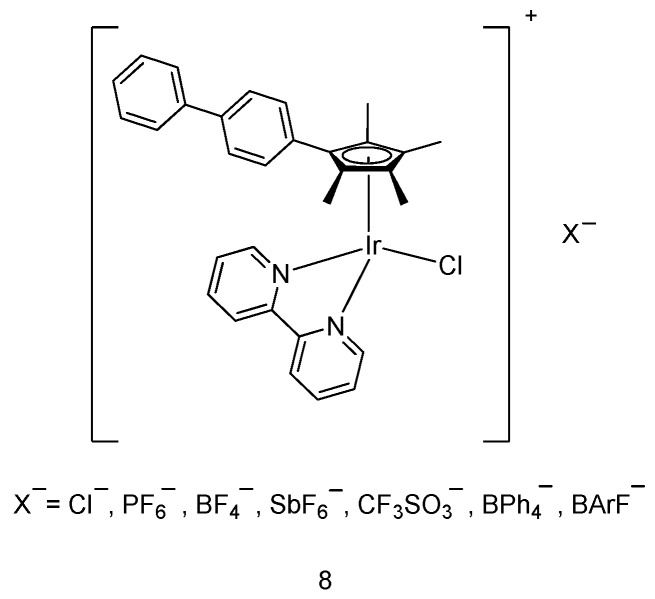
Varying the counteranionsin organometallic half-sandwich iridium(III) compounds.

Cuo, Liu and coworkers [35] have reported a group of organometallic half-sandwich iridium(III) complexes (**8**) bearing different counteranions (Figure 3). The cell viability of A549 human lung cancer cells was determined using 3-(4,5-dimethylthiazol-2-yl)-2,5-diphenyltetrazolium bromide (MTT) assay. The results indicated that smaller counteranions (Cl^−^, PF_6_^−^, BF_4_^−^, SbF_6_^−^, and CF_3_SO_3_^−^) provided good inhibitory effect against A549 cancer cells, with IC_50_ values of ≈10 μM. In contrast, larger counteranions (BPh_4_^−^, [3,5(CF_3_)_2_Ph]_4_B^−^, and BArF^−^) displayed insignificant cytotoxic effect. This study demonstrates that the nature of the counteranions can have a notable impact on the anticancer potency of the half-sandwich iridium(III) complexes. Flow cytometry experiments suggested that the changes in the mitochondrial membrane potential and ROS level played a crucial role in inducing the anticancer activity of these complexes.

**Figure 4:**
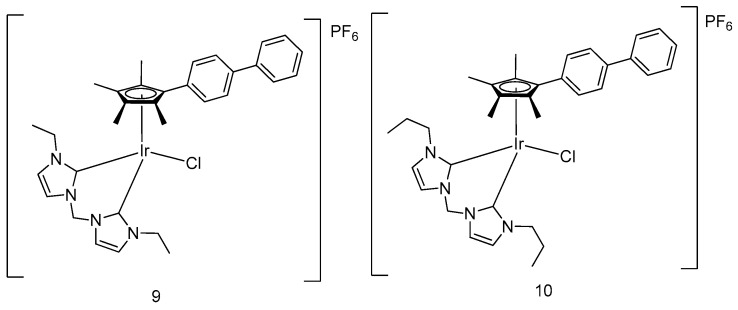
Organometallic half-sandwich iridium(III) compounds with N-heterocyclic carbene groups.

By incorporating various N-heterocyclic carbenes as C^C-chelating ligands into an iridium center, Liu and coworkers have devised a series of half-sandwich iridium complexes with improved antitumor potency [36]. Rather than through DNA binding, complexes **9** and **10** (Figure 4) were demonstrated to activate cell apoptosis via substantially increasing ROS levels in cells, rendering them as potent antitumor candidates. In that study, the effect of substituents was also investigated. For the complexes bearing the same N-heterocyclic carbene ligand, their anticancer activity (as measured based on the MTT assay) was enhanced in accordance with the increased number of substitutions in the pentamethylcyclopentadienyl motif. Additionally, the antitumor potency of the complex also hinged on the size of the hydrocarbon chain in the C^C ligand.

**Figure 5:**
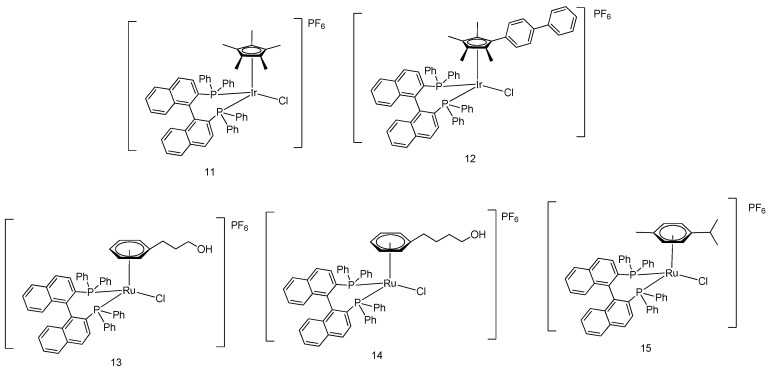
Organometallic half-sandwich iridium(III) and ruthenium compounds with P^P-chelating ligands.

The antitumor ability mechanism of cyclometalated half-sandwich iridium(III)-based complexes was further investigated by Liu and coworkers [37]. Five iridium- and ruthenium-based half-sandwich complexes **11**–**15** (Figure 5) with P^P-chelating ligands were synthesized for mechanistic study. All complexes exhibited antiproliferative behavior against A549 and HeLa cancerous cells. Complexes **11**–**15** displayed comparable anticancer activity to cisplatin against A549 cells and HeLa cells with IC_50_ values of 1.4–35.0 μM and 1.0–23.7 μM, respectively, as measured using the MTT assay. Cell cycle analysis indicated that the iridium and ruthenium complexes disturbed the cell cycle and caused cell apoptosis at the sub-G1/S phase and G1 phase, respectively. The results of the apoptosis assay and ROS induction assay revealed that the cytotoxicity of the complexes toward tumor cells is initiated from the elevation of cellular ROS level, possibly via the oxidation of NADH to NAD+ catalyzed by the half-sandwich metal complex, thereby causing cellular redox imbalance and apoptosis [38].

**Figure 6:**
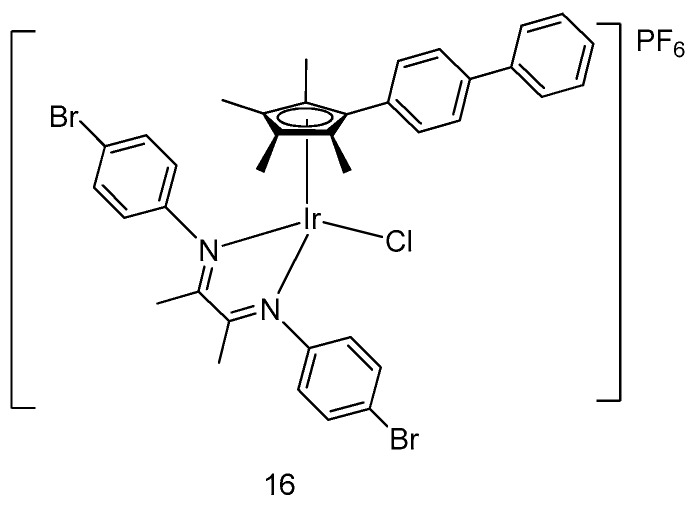
Organometallic half-sandwich iridium(III) complex **16**.

A novel half-sandwich iridium(III) complex **16** (Figure 6), containing α-diimine moiety as a N^N-chelating ligand, was devised by Liu and coworkers as a lysosome-targeted anticancer agent [39]. With a bromine substituent attached into the moiety, the electronic property of the metal center and the hydrophobicity of the complex was altered, which contributed to the distinctive antitumor property of the complex. Despite its higher cytotoxicity toward A549 and HeLa cancer cells than cisplatin as measured by the MTT assay, the complex was less selective and could also exert cytotoxic effect against normal cells (BEAS-2B). Furthermore, the anticancer activity of complex **16** was shown to originate from lysosomal membrane permeabilization, resulting in the elevation of ROS levels and the induction of apoptotic cell death.

**Figure 7:**
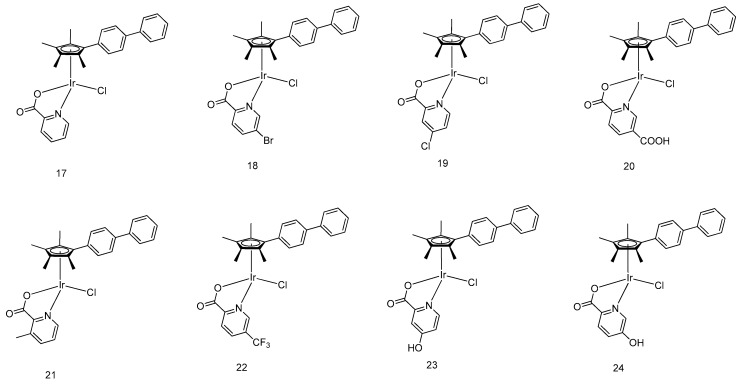
Organometallic half-sandwich iridium(III) with α-picolinic acid O^N-chelating ligands.

Moreover, eight half-sandwich iridium-based anticancer agents **17**–**24** (Figure 7) were synthesized by Liuand coworkers as novel anticancer agents [40]. Coordinated to an α-picolinic acid ligand, all complexes exhibited improved hydrophobicity and cytotoxicity toward A549 tumor cells based on MTT assay, with complex **22** bearing the most hydrophobic trifluoromethyl group being five times more cytotoxic toward cancer cells than cisplatin. The mechanistic study showed that those complexes could be delivered by serum albumin, and participate in the oxidation of NADH as a catalyst, resulting in drastic increases in cellular ROS levels. Through energy-dependent pathways, the complexes penetrates the tumor cell and selectively targeted mitochondria and lysosomes. Lysosomal damage accompanied by the fluctuation of mitochondrial membrane potential then leads to induced apoptotic cell death.

### 5.2. Development of Cyclometalated Octahedral Iridium(III) Compounds as Antitumor Agents

**Figure 8:**
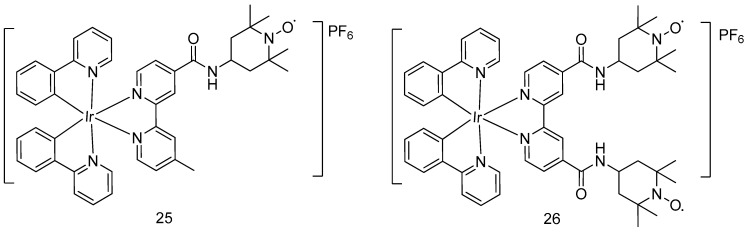
Octahedral iridium(III) compounds with 2,2,6,6-tetramethylpiperidine-N-oxyl (TEMPO) ligands.

Besides organoiridium(III) half-sandwich complexes, cyclometalated octahedral iridium(III) complexes have also been demonstrated in recent years to display noteworthy anticancer activity [41]. Very recently, Sadler et al. developed octahedral cyclometalated iridium(III) complexes bearing one (**25**) or two (**26**) 2,2,6,6-tetramethylpiperidine-*N*-oxyl (TEMPO) nitroxide spin labels, respectively (Figure 8). The bis-nitroxide complex **17** showed higher antitumor activity than the mono-nitroxide complex **25** in various cancer cell lines (e.g., A549 human lung cancer cells, PC3 prostate cancer cells, A2780 human ovarian cancer cells, and A2780Cis cisplatin-resistant human ovarian cancer cells). In the SRB assay, complex **26** demonstrated micromolar to submicromolar IC_50_ values in various cell lines with comparable or even strong potency compared to cisplatin (Table 1). That study provided evidence of the importance of the TEMPO radical in conferring inhibitory activity against cancer cells. In addition, these complexes were observed to localize in the mitochondria of the PC3 cells, leading to changes in the membrane potential of mitochondria in cancer cells as detected by flow cytometry. This suggests that the compounds could be acting via targeting mitochondrial apoptosis to exert their anticancer activities.

**Figure 9:**
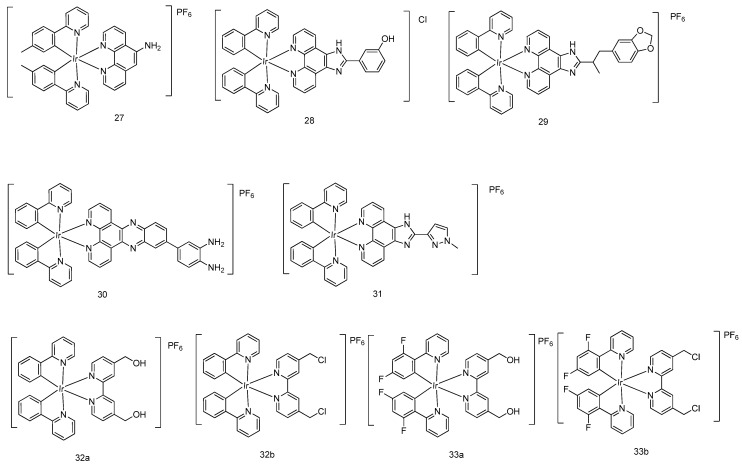
Various cyclometalated octahedral iridium(III) compounds.

Lu, Liu, Chao and coworkers recently developed a cyclometalated iridium(III) complex **27** with antibacterial and anticancer activity (Figure 9) [42]. In the MTT assay, complex **27** significantly inhibited cancer cell growth at 10 μM in A2780 human ovarian cancer cells, SKOV3 human ovarian cancer cells, HeLa human cervical cancer cells, A2058 Caucasian metastatic melanoma cells, and A375 human melanoma cells.

Wang and coworkers developed the cyclometalated iridium(III) complex **28** [Ir(ppy)_2_(HPIP)]Cl (where HPIP = 2-(2-hydroxyphenyl)imidazo[4,5-f]1,10-phenanthroline), which displayed stronger cytotoxic potency compared with cisplatin in various cancer cell lines, including the cisplatin-resistant A549 cells where it showed 10 times higher cytotoxicity (IC_50_ of **28** = 5.2 μM) than cisplatin as revealed by the MTT assay [43]. Further experiments showed that complex **28** significantly changed the membrane potential of mitochondria, suggesting that it acted via inducing apoptotic cell death through the intrinsic mitochondria-mediated pathway.

A range of iridium(III) polypyridyl compounds, including [Ir(ppy)_2_(BDPIP)]PF_6_ [BDPIP = 2-(1-benzo[d]dioxol-5-yl)propan-2-yl)-1H-imidazo[4,5-f] [1,10]phenanthrene] (**29**) [44], [Ir(ppy)_2_(DPBD)]PF_6_ [DPBD = 4-(dipyrido[3,2-*a*:2′,3′-*c*]phenazin-11-yl) benzene-1,2-diamine] (**30**) [45], and [Ir(ppy)_2_(MHPIP)]PF_6_ [MHPIP = 2-(1-methyl-1*H*-pyrazol-3-yl)- 1*H*-imidazo[4,5-f][1,10]phenanthroline] (**31**) [46], were reported as potent anticancer agents. Complex **29** displayed IC_50_ values in the micromolar range in various cancerous cells as revealed by the MTT assay, including SiHa human cervical cancer cells, SGC-7901 human gastric cancer cells, BEL-7402 human liver cancer cells, HepG2 human liver cancer cells, A549 human lung cancer cells, and HeLa human cervical cancer cells. Investigation into the cell death pathway showed that complex **29** induced a dose-dependent reduction of mitochondrial membrane potential in A549 cells. Compared to the distinguished anticancer activity of complex **29**, complex **30** with 2-(1*H*-imidazol-2-yl) pyridine ligand is less cytotoxic toward those cancer cells.

Tan, Mao and coworkers discovered four mitochondria-targeted luminescent cyclometalated iridium(III) complexes (**32a,b** and **33a,b**) as potent anticancer therapeutics [47]. Notably, complexes **32b** and **33b** containing chloromethyl groups in the N^N ligand displayed much higher inhibitory effects in A549R cisplatin-resistant human lung cancer cells (IC_50_ was 0.64 and 0.74 µM for complex **32b** and **33b**, respectively, by the MTT assay) than complexes **32a** and **33a** with hydroxymethyl groups. The mitochondrial immobilization assay revealed that complexes **32** and **33** exerted their anticancer activities by localizing in the mitochondria through targeting mitochondrial metabolism.

**Figure 10:**
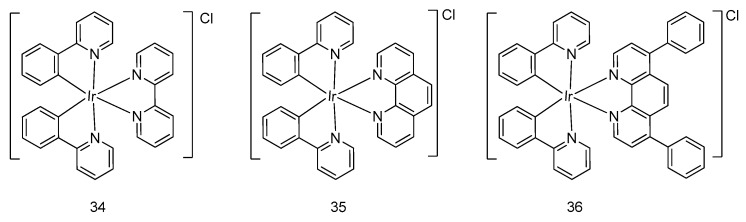
Cyclometalated octahedral iridium(III) compounds with 1-phenyl-pyridine C^N ligand and different N^N ligands.

Zhou, Fei and coworkers developed three iridium(III) complexes **34**–**36** (Figure 10) with different N^N ligands, including 2,2′-bipyridine, 1,10-phenanthroline, and 4,7-diphenyl-1,10-phenanthroline, to examine the structural effect of auxiliary ligands on their antitumor activity [48]. Although all complexes exhibited remarkable antitumor activity in the MTT assay, their photophysical property and anticancer potency were enhanced when the molecular weight of the N^N auxiliary ligand increased. Remarkably, the preferential accumulation of iridium(III) complex **36**, with distinctive cytotoxicity and cellular uptake efficiency in the endoplasmic reticulum, was observed, which could subsequently induce mitochondria-mediated apoptosis by causing noticeable fragmentation and swelling of mitochondria in HeLa cells with a lower IC_50_ value compared with cisplatin.

**Figure 11:**
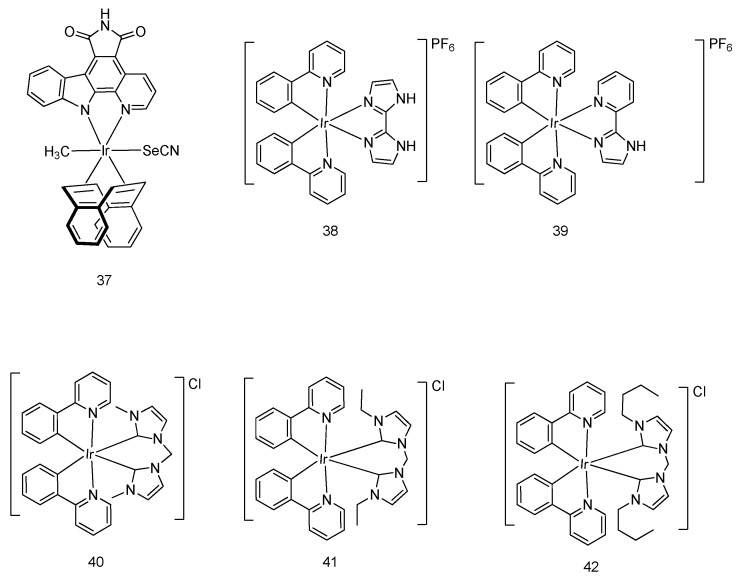
Diverse cyclometalated octahedral iridium(III) compounds.

An iridium complex **37** (Figure 11) was devised by Meggers and coworkers as a phototoxic anticancer agent [49]. Upon irradiation by visible light, the cellular survival of HeLa cells was reduced to 12.5% in the presence of complex **37** as determined by the MTT assay, revealing that the complex could effectively trigger cellular apoptosis. Meanwhile, insignificant photo-induced cytotoxicity was observed in the dark. The mechanism behind photo-induced anticancer activity of the complex has not yet been discovered. Interestingly, complex **37** also exhibited light-independent antitumor activity, owning to its ability to suppress VEGFR3 activity [50].

Two cyclometalated iridium complexes **38** and **39** (Figure 11) were developed by Chen, Tan, Mao and coworkers [51]. With a 2,2′-biimidazole N^N ligand functionalized for anion binding, the iridium complex **38** served as an anion transporter to regulate lysosomal pH through hydrogen transfer, resulting in autophagic flux suppression. Importantly, those complexes were shown to prompt apoptotic cell death through the increase of cellular ROS levels. In the MTT assay, the complexes were active against various kinds of cancer cells, including MDA-MB-231 human metastatic breast cancer cells, HepG2 human hepatoma cells, A549R cisplatin-resistant cells, A549 human lung adenocarcinoma cells, and HeLa human cervical cancer cells.

Three iridium complexes **40**–**42** (Figure 11) containing N-heterocyclic carbene ligands were reported by Mao and coworkers [52]. These complexes showed remarkable cytotoxicity by the MTT assay, and also induced apoptotic cell death through mitochondrial dysfunction, in which the anti-apoptotic protein Bcl-2 expression was inhibited and cyt-c was released. Importantly, complexes **40**–**42** exhibited higher cytotoxicity toward various human cancer cells than cisplatin, including HeLa, A549, A549R, HepG2, MCF-7, and LO2, without showing disruption of the cell cycle and genotoxicity. Notably, upon light illumination, the IC_50_ value of the complexes decreased by 9- to 3488-fold compared with the dark environment, indicating that anticancer potency is dramatically enhanced.

## 6. Conclusions

In this review, we summarized the latest advances in developing half-sandwich and octahedral iridium(III)-centered metal complexes as anticancer agents, with a particular focus on those targeting apoptotic cell death in cancers. Iridium(III) complexes display a good combination of high biological activity, good cell permeability, and high stability in living cells. The distinct structure and properties of half-sandwich and octahedral iridium(III) complexes in these studies are interesting and give significant prospects for the future application of iridium(III) complexes as anticancer agents.

Mitochondria-targeted apoptosis represents a central checkpoint during the apoptosis process by integrating several different exogenous/endogenous signals from nearby organelles such as autophagic vacuoles, lysosomes, the cytosol, or the nucleus. Cross-talk within distinct organelles within the cellular environment is inevitable and plays a crucial role in determining cell fate through the activation of favorable lethal or survival signaling pathways. This interorganelle level-based communication can be regulated by a range of biochemical reactions such as (de)phosphorylation, proteolysis, and translocation of proteins, redox reactions, ion/metabolites fluctuations, or transcription processes. Interestingly, the majority of mitochondria-targeted apoptosis processes directly respond to the two predominant signal regulation mechanisms based on caspases or matrix metalloproteinases, with a subsequent cascade activation of other surrounding organelles including the endoplasmic reticulum, Golgi apparatus, lysosomes, or the nucleus.

For developing highly selective mitochondria-targeted anticancer therapies, a detailed understanding of the differences between various mechanisms and the dynamic variation of biochemical reactions during mitochondria-targeted apoptosis is necessary. The development of novel drugs can be inspired from existing conventional mitochondrial-targeted anticancer agents. On the one hand, a high selectivity of the anticancer drugs can be achieved by improving their cytotoxic activity against malignant cells over healthy cells. On the other hand, selectivity toward the specific mitochondria organelles over other cross-talking organelles should also been taken into consideration to facilitate the drug discovery process. Considering the intrinsic cytotoxicity activity generated from transition metal centers, unnecessary side effects may also hinder the further application of metal-conjugated anticancer therapies. Given the ease of modifying inorganic metal complexes, various potent metal-based complexes with diverse biological activities can be obtained by the modification of chelating ligands. In comparison with purely organic anticancer molecules, transition metal complexes, and particularly iridium(III) complexes, have remarkable advantages in exerting mitochondria-targeted anticancer activity with the conjugation of targeted binding moiety within the scaffolds of the iridium(III) complexes. Today, drug resistance against conventional anticancer drugs has aroused significant attention. The development of iridium(III)-based complexes offers the opportunity to bypass this resistance and improve the efficiency of killing the targeted cancerous cells. In this scenario, the development of either the half-sandwich or cyclometalated octahedral iridium(III) complexes targeting mitochondrial apoptosis may therefore serve as a useful strategy for combating cancerous cells.

## Figures and Tables

**Table 1:** IC_50_ values for complexes **25** and **26**, and cisplatin against A2780 human ovarian, A2780Cis cisplatin-resistant human ovarian, A549 human lung, and PC3 human prostate cancer cell lines.

Cell Lines		IC_50_/μM	
	25	26	Cisplatin
A2780	14.5 ± 0.5	3.0 ± 0.2	1.2 ± 0.2
A2780Cis	8.6 ± 0.1	2.57 ± 0.08	13.4 ± 0.3
A549	13.8 ± 0.5	7.5 ± 0.6	3.2 ± 0.1
PC3	16.21 ± 0.08	0.53 ± 0.02	4.1 ± 0.5
